# The Role of Surface Chemistry and pH Shifts in the Sorption of Common NSAIDs on Coconut and Orange Waste-Derived Carbon Materials

**DOI:** 10.3390/molecules31183209

**Published:** 2026-09-11

**Authors:** Jorge A. Olivarez, Viridiana Hernández, Hana P. Mandujano-Zúñiga, Romary A. Sánchez, Luis A. Godínez, Josué D. García-Espinoza, Alina Z. Vela-Carrillo, Francisco J. Rodríguez-Valadez, Monserrat Santos-Blanco, Raúl Ortega-Borges, Irma Robles

**Affiliations:** 1 Centro de Investigación y Desarrollo Tecnológico en Electroquímica S. C., Parque Tecnológico Querétaro, Sanfandila, Pedro Escobedo 76703, Querétaro, Mexico; jolivarez@cideteq.mx (J.A.O.); frodriguez@cideteq.mx (F.J.R.-V.); msantos@cideteq.mx (M.S.-B.);; 2 Centro de Investigación en Química para la Economía Circular, CIQEC, Facultad de Química, Universidad Autónoma de Querétaro, Centro Universitario, Santiago de Querétaro 76010, Querétaro, Mexico; luis.godinez@uaq.mx (L.A.G.); josue.daniel.garcia@uaq.mx (J.D.G.-E.)

**Keywords:** agroindustrial-derived activated carbon, pharmaceutical sorption, surface chemistry, pH shift, coconut shell, orange peel

## Abstract

This study investigated the sorption of three widely detected non-steroidal anti-inflammatory drugs, naproxen, diclofenac, and ibuprofen, onto biochars and activated carbons derived from coconut shell and orange peel. Activated carbons were prepared under chemical activation with ZnCl_2_ and H_3_PO_4_. The sorbent materials were characterized in terms of surface functional groups, acid–base properties, and pzc, while the pH was monitored along the sorption experiments. Results suggest that the activating agent played an important role in controlling surface chemistry and acid–base properties; ZnCl_2_-activated carbons exhibited near-neutral pzc values and a balanced distribution of functional groups, while H_3_PO_4_-activated materials showed highly acidic surfaces with lower pzc. The solution pH evolved during sorption instead of remaining at its initial value. The sorption performance showed a clear dependance on surface chemistry, ZnCl_2_-activated carbons exhibited a superior sorption capacity as well as faster kinetics under neutral conditions, this behavior may be associated to reduced electrostatic repulsion toward anionic model molecules. Under acidic conditions, some H_3_PO_4_-activated materials showed comparatively favorable sorption behavior, consistent with possible contribution from hydrogen bonding and hydrophobic interactions. Kinetic analysis showed time-dependent uptake profiles consistent with more than one mass-transfer contribution; however, no single rate-controlling mechanism could be established from the available data. The results indicate that NSAID sorption under the investigated conditions was associated with the surface acid–base properties of the carbon materials, the initial-to-final pH shifts, and the pH-dependent speciation of the pharmaceuticals. These findings provide an empirical basis for evaluating the performance of agroindustrial waste-derived carbon materials toward ionizable pharmaceuticals and highlight the importance of considering both surface chemistry and the measured solution pH when interpreting sorption behavior.

## 1. Introduction

The widespread occurrence of pharmaceutical compounds in aquatic environments has emerged as a critical environmental concern over the past two decades. Among these contaminants, non-steroidal anti-inflammatory drugs (NSAIDs) such as naproxen, ibuprofen, and diclofenac are frequently detected in surface waters, wastewater effluents, and even drinking water sources due to their extensive consumption and incomplete removal in conventional treatment systems [1]. Their persistence, bioactivity, and potential ecotoxicological effects have raised increasing concern regarding long-term environmental and human health risks [2,3].

Conventional wastewater treatment processes are not specifically designed to remove micropollutants, leading to the continuous release of these compounds into aquatic ecosystems [4,5]. As a result, advanced treatment technologies have been widely investigated, among which sorption onto carbonaceous materials remains one of the most effective and economically viable approaches due to its simplicity, high efficiency, and scalability [6].

In recent years, there has been growing interest in the development of sustainable sorbents derived from agroindustrial residues, such as coconut shell and fruit peels, which offer a low-cost and environmentally friendly alternative to commercial activated carbons. These materials can be transformed into biochars or activated carbons with tailored physicochemical properties through thermochemical and chemical activation processes [7,8]. In particular, activating agents such as phosphoric acid (H_3_PO_4_) and zinc chloride (ZnCl_2_) are known to significantly influence pore development and surface chemistry, resulting in materials with distinct sorption behaviors [9,10].

Despite extensive research on the sorption of pharmaceuticals onto activated carbons, most studies have focused primarily on equilibrium capacities and isotherm modeling, often overlooking the fundamental role of surface chemistry and acid–base interactions. Parameters such as the distribution of oxygen-containing functional groups and the point of zero charge (pzc) are known to strongly affect sorption mechanisms, especially for ionizable compounds [11,12]. However, these parameters are rarely integrated into a comprehensive framework that links material properties with sorption performance.

Furthermore, sorption studies are typically conducted under controlled pH conditions, assuming that the solution pH remains constant throughout the experiment. This assumption is particularly problematic for systems involving carbonaceous materials, where surface functional groups can induce significant pH variations through proton exchange and apparent acid–base buffering effects [13,14]. For ionizable pharmaceuticals such as NSAIDs, whose speciation strongly depends on pH, such dynamic changes can alter sorption mechanisms and lead to misinterpretation of experimental results [15,16,17].

Recent studies have highlighted the importance of pH-dependent speciation in sorption processes; however, the interplay between surface chemistry, pH evolution, and sorption performance remains insufficiently understood [18]. In particular, there is a lack of systematic studies that simultaneously evaluate (i) different precursors, (ii) different activation methods, (iii) surface functional groups, and (iv) sorption behavior under dynamically evolving pH conditions.

In this context, the present work aims to bridge this gap by investigating the sorption of naproxen, ibuprofen, and diclofenac onto biochars and activated carbons derived from coconut shell and orange peel, prepared via ZnCl_2_ and H_3_PO_4_ activation. Unlike conventional approaches, this study integrates surface chemistry characterization, pzc analysis, sorption experiments, and kinetic evaluation within a unified framework that explicitly considers pH drift during the process. By examining the relationships among surface acid–base properties, PZC, pH conditions, and sorption behavior, this study aims to identify empirical trends that may contribute to understanding the performance of biomass-derived carbon materials toward ionizable pharmaceuticals.

## 2. Results and Discussion

### 2.1. Effect of Surface Chemistry

The surface chemistry of the prepared carbons exhibited a strong dependance on both precursor material and activating agent, as evidenced by Boehm titration and pzc measurements in Table 1. It is possible to see that biochars (BC and BO) showed a predominance of carboxylic groups accompanied by lower concentrations of phenolic and lactone groups; this distribution resulted in pzc values between 7.2 and 8.0, indicating near-neutral to slightly basic surfaces as a result of the surface groups and the carbonous structure, as shown in Table 1.

In contrast, ZnCl_2_-activated carbons (CZ and OZ) displayed a more balanced distribution of functional groups, compared to those activated with H_3_PO_4_. These materials exhibited pzc values close to neutrality, from 6.8 to 7.5, that suggest a chemically stable interface under environmental conditions.

On the other hand, the commercial activated carbon (CAC) exhibited a pzc of 5.7, with a balanced acid–basic surface chemistry compared to activated carbons from coconut shells and orange peel. This intermediate acid–basic character explains its relatively stable sorption performance across different pH conditions and supports its role as a suitable reference for evaluating agroindustrial-derived activated carbons.

Conversely, H_3_PO_4_-activated carbons (CP and OP) showed a marked different chemical behavior characterized by a higher acidity, up to 5.3 mmol/g, predominantly given by lactone groups, and a drastic reduction in phenolic content, and this property led to very low pzc values, from 1.4 to 2.0, indicative of a strongly acidic surface.

These observations are consistent with previous studies that reported phosphoric acid activation promotes the formation of phosphate esters and oxygenated acidic groups, whereas ZnCl_2_-activation favors the development of aromatic structures and a relatively more neutral surface chemistry [9,10,19,20,21,22].

A decreasing tendency between total acidity and pzc was observed (Table 1) that suggests surface oxygenated groups are among the major factors governing pzc, together with mineral matter and carbon structure. Similar observations have been reported for lignocellulosic carbonaceous materials, where increasing oxygenate functionalities leads to a decrease in pzc and enhanced surface polarity [23,24,25].

These differences demonstrate distinct surface acid–base characteristics among the carbon materials. PZC is used here as an acid–base surface descriptor; however, because zeta potential was not measured, the effective surface charge under the individual sorption conditions cannot be directly established.

### 2.2. Dynamic pH Effect and Sorption Performance

**Figure 1:**
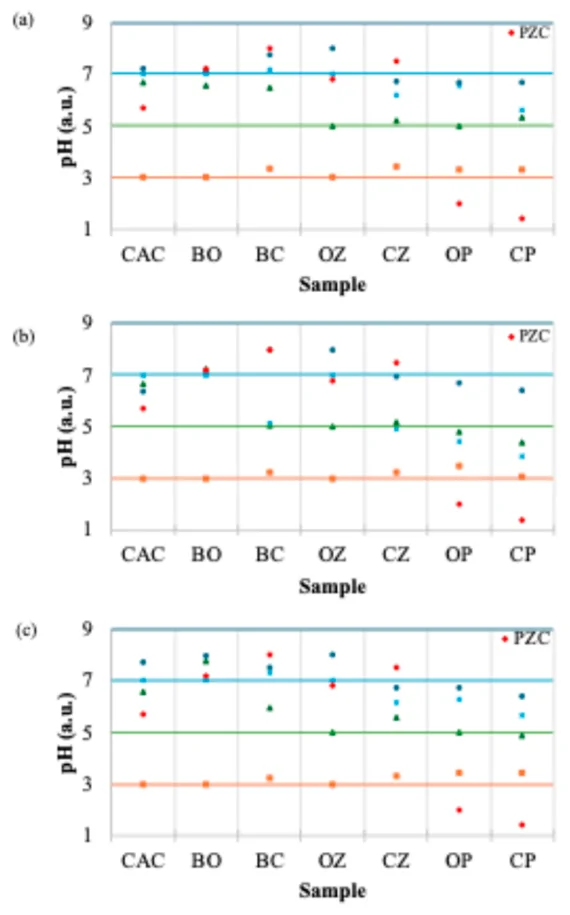
A comparison of initial and final pH in relation to the PZC of the carbon materials during sorption experiments. Initial pH (represented by colored lines) and final pH after sorption for all materials (represented by colored symbols); orange: pH 3, green: pH 5, light blue: pH 7, dark blue: pH 9. The distribution of final pH values across materials, (**a**) naproxen, (**b**) ibuprofen, and (**c**) diclofenac.

Figure 1 shows the behavior of pH and pzc comparatively for sorbent materials and model molecules; the sorption experiments showed that the system is governed by a dynamic acid–base equilibrium rather than a fixed pH condition. Only the strong acidic condition (pH 3) remains relatively stable, with final values between 3 and 3.6. Biocarbon samples (samples at pH 5 and 7) predominantly converge toward a neutral pH that can be attributed to the buffering capacity of the sample given by acidic and basic groups. It was also possible to see that materials with pzc values near neutrality, ZnCl_2_-activated carbons and biochars, exhibited a comparatively pronounced initial to final pH shifts toward near-neutral values. The commercial activated carbons (CAC) showed an intermediate initial to final pH response relative to the biomass derived materials that is consistent with its intermediate pzc (5.7), although these materials resulted less stable compared to ZnCl_2_-activated carbons.

In contrast, the H_3_PO_4_-activated materials exhibited different initial to final pH shifts from those observed for the ZnCl_2_-activated carbons and biochars. These differences are consistent with their markedly different surface acid–base properties, although the present measurements do not identify the individual proton-transfer or surface reactions responsible for the observed pH changes. The behavior is compatible with proton exchange and phosphate surface functionalities generated during activation [9,10]. Similar pH drift phenomena have been reported for carbonaceous material, that are commonly attributed to surface complexation reactions and to ion exchange processes involving surface functional groups such as -COOH and -CO [26,27,28]. This is a central governing factor in the sorption behavior that has been studied by our group [19].

The sorption experiments in this study conducted at an initial fixed pH do not necessarily represent the natural conditions under sorption processes occur; therefore, interpretation of the sorption performance must consider the real equilibrium pH, particularly when dealing with ionizable compounds such as naproxen, diclofenac, and ibuprofen, common NSAIDs.

Because NSAID ionization depends on solution pH, both pH_i_ and pH_f_ were considered when interpreting sorption behavior. In particular, the relationship between the measured pH values and the corresponding pharmaceutical pKa provides an estimate of the relative abundance of protonated (HA) and deprotonated (A^−^) species under the experimental conditions.

For all sorbent–pharmaceutical systems, an operational equilibrium condition was identified when the measured pharmaceutical concentration no longer changed with further contact time.

**Figure 2:**
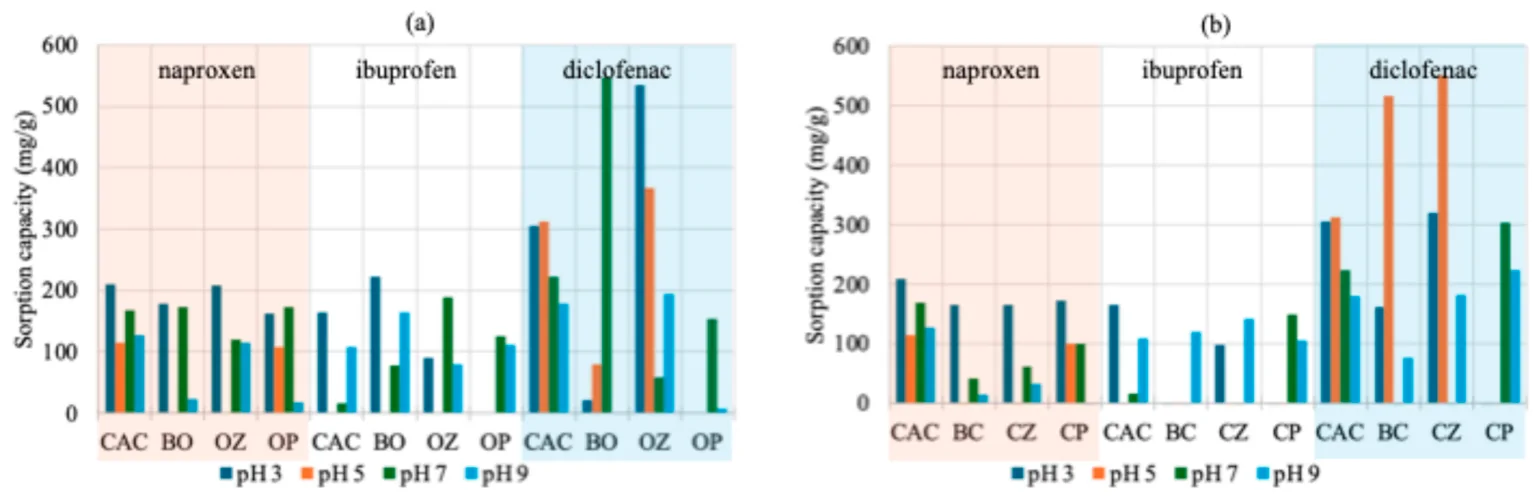
Sorption performance under varying pH conditions. Heatmap representation of sorption capacity (mg g^−1^) of naproxen, ibuprofen, and diclofenac across different initial pH values and materials derived from (**a**) orange peel (BO: orange peel biocarbon, OZ: ZnCl_2_-activated carbon from orange peel, OP: H_3_PO_4_-activated carbon from orange peel) and (**b**) coconut shell (BC: coconut shell biocarbon, CZ: ZnCl_2_-activated carbon from coconut shell, CP: H_3_PO_4_-activated carbon from coconut shell).

**Figure 3:**
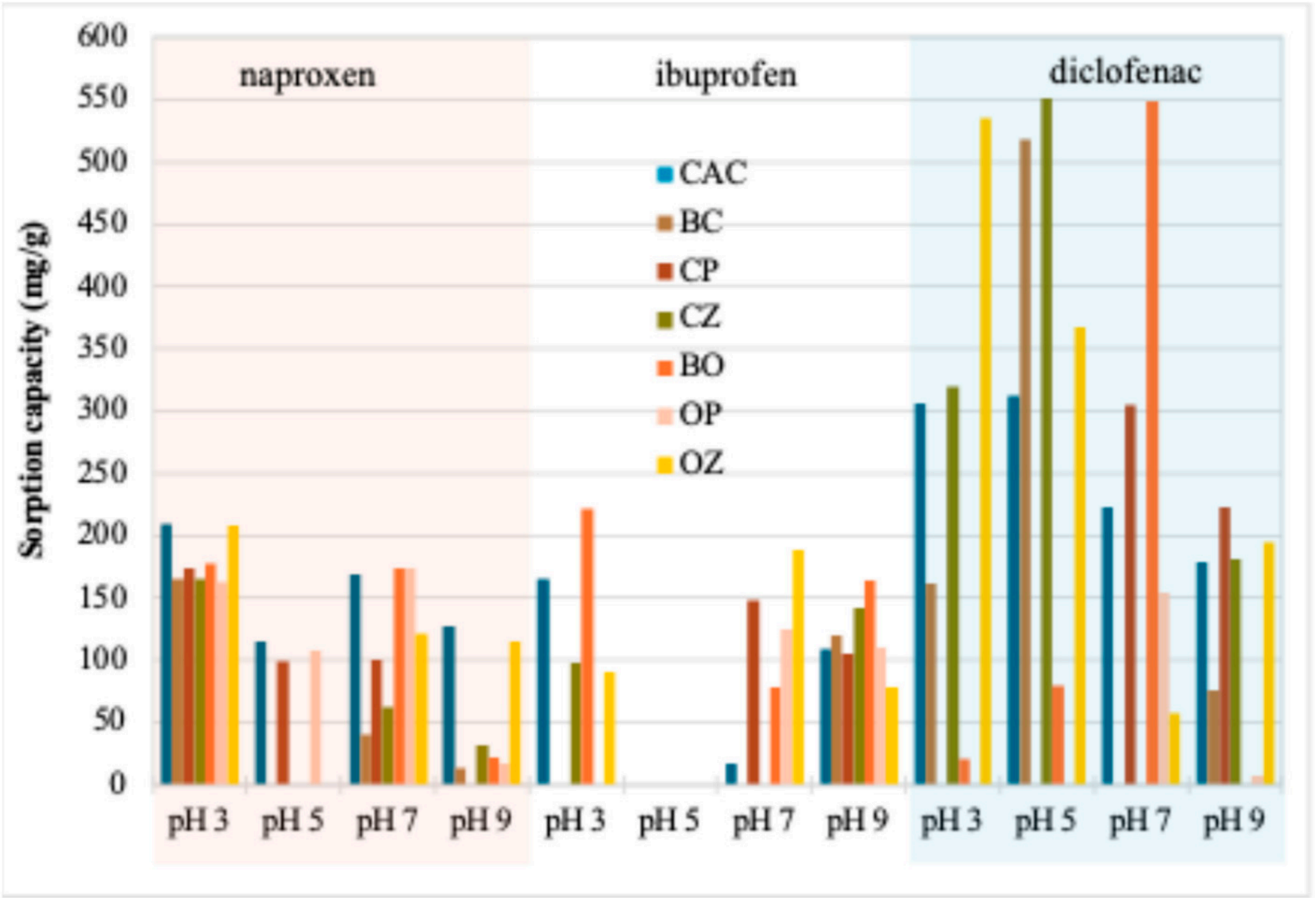
Sorbent behavior across pH conditions for naproxen, ibuprofen, and diclofenac, comparison of ZnCl_2_-activated carbons and H_3_PO_4_-activated materials.

Sorption results were analyzed during the sorption processes, considering an initial fixed pH and the final pH given by the parametric correlation among sorbent and model molecules. From the results in Figure 2 and Figure 3, we identify two distinct regimes: (1) the acidic regime (pH 3 to 3.6), and (2), the neutral regime for pH from 6.7 to 7.5, associated mainly with experiments initiated at pH 3, and a near-neutral range, observed for several systems initiated at higher pH values. These ranges are used descriptively to facilitate comparison among the sorbents.

Under acidic conditions, naproxen, diclofenac, and ibuprofen are partially protonated, this condition enhances hydrophobic interactions and hydrogen bonding with the surface of sorbent materials. In this regard, H_3_PO_4_-activated carbons showed comparatively high sorption uptake under the experimental conditions evaluated due to the high density of oxygenated functional groups that may act as hydrogen bond donors or acceptors [29].

On the other hand, at neutral conditions, a more representative scheme for environmental at natural conditions, the pharmaceuticals naproxen, diclofenac, and ibuprofen exist predominantly in their anionic form [30]. Under near-neutral conditions, some ZnCl_2_-activated carbons exhibited comparatively high NSAID uptake. Their PZC values were also closer to the measured solution pH than those of the H_3_PO_4_-activated materials. This association suggests that surface acid–base properties may contribute to the observed differences in sorption behavior. However, because zeta potential was not measured, the magnitude and sign of the effective surface charge under each sorption condition cannot be directly established, and electrostatic interactions cannot be isolated as the controlling contribution [31].

Previous reports on NSAID sorption reported that the sorption of naproxen and diclofenac is strongly influenced by π–π interactions and hydrophobic effects, particularly on carbon materials with developed aromatic structures [32,33,34].

Moreover, we observed this tendency of sorption capacity: diclofenac > naproxen > ibuprofen. This is consistent with their molecular structure and hydrophobicity, as diclofenac contains two aromatic rings and exhibits higher log K_ow_ values [35], enhancing its affinity for carbonaceous surfaces.

These results highlight that sorption performance is not solely determined by surface area or porosity, it is also influenced by complex interactions between surface chemistry, pH, and sorbate speciation.

### 2.3. Time-Dependent Sorption Behavior Under Initial pH Conditions

The observed time-dependent sorption profiles revealed that for most materials and model molecules, most sorption systems exhibited high uptake by the first experimental sampling point (0.5 h). Because no measurements were collected before 0.5 h, the initial uptake region was not experimentally resolved. Consequently, no specific mass-transfer mechanism is assigned to the period preceding the first experimental measurement. This rapid uptake was consistently observed across naproxen, ibuprofen, and diclofenac systems, regardless of the initial pH conditions. Such behavior suggests that the sorption is initially dominated by readily accessible surface sites and strong interactions between the sorbate molecules and the material surface.

As a consequence, empiric classic kinetic models, including pseudo-first order and pseudo-second order, did not provide satisfactory fits to the experimental data. The pseudo-first order model systematically underestimated the equilibrium sorption capacity, while the pseudo-second order model, although occasionally yielding acceptable correlation efficiencies, produced inconsistent kinetic parameters across different pH conditions and materials. These deviations are attributed to initial to final pH shift, surface heterogeneity, and simultaneous mass-transfer steps. The fitted kinetic parameters should therefore be regarded as apparent empirical descriptors of the measured time range rather than intrinsic mechanistic constants.

These findings are consistent with previous reports indicating that classical kinetic models may be inadequate for describing the sorption processes occurring under rapidly changing physicochemical conditions or involving highly heterogeneous surfaces.

#### 2.3.1. Weber–Morris Analysis of the Experimental Uptake Profiles

The Weber–Morris representation was examined to evaluate changes in the apparent uptake behavior over the measured experimental time range. Several profiles showed multilinear behavior, indicating that a single linear relationship with t^1/2^ was insufficient to describe the complete dataset. However, because the first measurement was obtained at 0.5 h, the initial uptake region was not experimentally resolved. Consequently, the individual linear regions should not be interpreted as direct evidence of specific sequential mass-transfer mechanisms. Changes in slope observed in some Weber–Morris plots are therefore discussed descriptively and may reflect changes in the relative importance of different transport contributions during the measured time range.

From the results, it is possible to suggest that the sorption process is given by a combination of external mass transfer and internal diffusion processes.

While ZnCl_2_-activated carbons exhibited higher intraparticle diffusion rate constants (k_id_), comparatively, H_3_PO_4_-activated carbons showed lower diffusion rates, likely due to the presence of a higher density of oxygenated functional groups [19] that for this process may hinder mass transfer or even introduce additional interaction barriers [36]. These results suggest that sorption kinetics are controlled by a multi-stage diffusion process rather than a single kinetic regime.

Previous characterization of these biomass-derived activated carbons indicates that chemical activation modifies their textural and morphological properties [9,10]. These previously reported characteristics may contribute to the differences in sorption observed here; however, their specific contribution to NSAID uptake cannot be isolated from the present dataset. Aromatic carbon domains can potentially participate in π–π interactions with aromatic NSAIDs, as reported for carbonaceous adsorbents in the literature [11,12]. However, the extent of aromatic ordering and its contribution to sorption were not directly evaluated in the present study.

#### 2.3.2. Influence of Surface Chemistry on Kinetic Behavior

The results of the sorption process indicate that the kinetic behavior strongly depended on the surface chemistry of the sorbent materials, which is govern by both the precursor and the activating agent.

ZnCl_2_-activated carbons consistently exhibited faster sorption rates and a more pronounced diffusion-controlled behavior; this process can be attributed to the corresponding surface chemistry, near-neutral pzc, which may facilitate a rapid transport of sorbate molecules into the internal structure of the material.

The higher uptake observed for some ZnCl_2_-activated carbons may reflect a combination of surface chemical and textural characteristics. Although textural properties of these materials have been characterized previously [9], the present experiments do not allow for their individual contribution to NSAID uptake to be quantified.

In contrast, H_3_PO_4_-activated carbons showed slower sorption kinetics and a better fit to Elovich model, indicating a heterogenous sorption process with a wide distribution of activation energies. The high density of acidic functional groups in these materials likely leads to stronger but more localized interactions, resulting in slower overall sorption rates.

Biocarbons, on the other hand, exhibited intermediate behavior, characterized by moderate sorption rates and significant surface heterogeneity that is consistent with the high phenolic content and a less developed pore structure, given by the intrinsic properties of precursors.

These global findings highlight that surface chemistry not only determines sorption capacity but also promotes a specific kinetic performance, influencing both the rate and the sorption process.

#### 2.3.3. Effect of Dynamic pH on Sorption Process

From this study, it was possible to identify that the comparison of pH_i_ and pH_f_ during the sorption process was a key factor that influenced the sorption behavior. As described in Section 2.2, the pH solution tends to shift toward near neutral values regardless of the initial conditions, particularly for systems initially set at pH 5 and 9. This pH drift induces significant changes in both the surface charge of the sorbent material and the speciation of model molecules, which are weal acids with pKa values in the range of 4 and 5. As a result of this study, a suggested dominant sorption mechanism evolves over time, transitioning from hydrophobic and hydrogen bonding interactions at lower pH to electrostatic and π–π interactions. Under near-neutral conditions, π–π interactions between aromatic NSAID moieties and carbon domains represent a plausible contribution to sorption; however, this interaction was not directly identified experimentally in the present study. At acidic pH, the greater proportion of protonated species may favor non-electrostatic interactions, potentially including hydrogen bonding with oxygen-containing surface functionalities. The present data, however, do not allow for the contribution of hydrogen bonding to be quantified independently.

This study did not focus on a chemistry stationary system, which helped to study the intrinsic variability and the parametric interactions along the sorption process. In this sense, this study showed that the sorption process is given by a time dependent interplay between surface chemistry, solution chemistry, and mass-transfer processes. Therefore, the observed deviations from traditional kinetic models are not merely fitting limitations but reflect the intrinsic complexity of the system.

In this regard, this study suggests that sorption kinetics in agro-waste-derived carbons cannot be adequately described by conventional single mechanisms models. Instead, the process should be interpreted as a multi-mechanistic process that involves the following:

1. A rapid surface sorption;
2. Intraparticle diffusion;
3. Dynamic changes in physicochemical conditions.

Among the preliminary evaluated models, the Weber–Morris model provided the most meaningful insight into the sorption process, while the Elovich model showed the heterogeneity of the surface of specific cases; however, no single model was sufficient to fully describe the system.

These findings emphasize that kinetic modeling should be used as an analysis tool rather than a purely predictive one, particularly for systems involving complex materials and dynamic environmental conditions. Specifically, in this study, the kinetic analysis reveals that the sorption of naproxen, diclofenac, and ibuprofen onto carbon materials derived from agroindustrial residues was governed by a dynamic and a multi-stage process, strongly influenced by surface chemistry and comparison of initial and final pH values. This approximation shows the need to move beyond classical kinetic models and adopt more comprehensive approaches that account for the coupled effects of mass transfer, surface heterogeneity, and acid–base interactions.

To further support the observed kinetic behavior, a comparative analysis of kinetic models across all materials, model molecules, and pH conditions is presented in Table 2. This analysis highlights the dominant sorption regimes and their relationship with surface chemistry and experimental conditions.

### 2.4. Association Between Surface Acid–Base Properties and Apparent Kinetic Behavior

**Figure 4:**
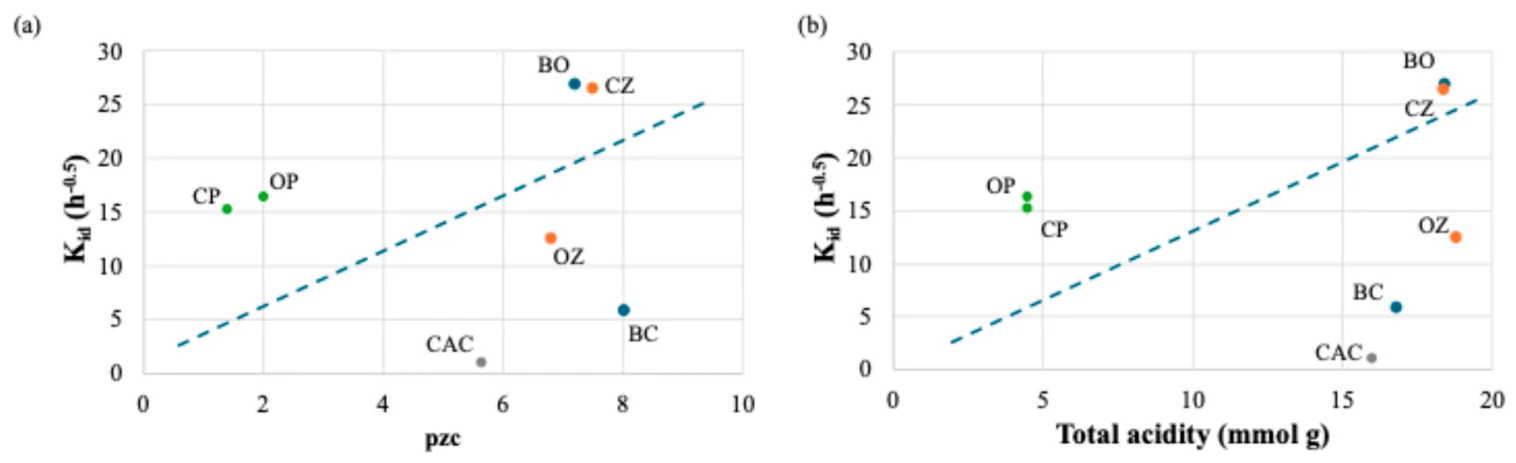
Correlation between intraparticle diffusion rate constant (k_id_) and surface properties of the carbon materials: (**a**) kid as a function of pzc and (**b**) kid as a function of total acidity. Data correspond to averaged values per material, considering only physically meaningful kid values from the second Weber–Morris region. Color coding: green (H_3_PO_4_-activated carbons), orange (ZnCl_2_-activated carbons), blue (biochars), and gray (commercial activated carbon, CAC).

To elucidate the parametric influence on the sorption process, Figure 4 shows the association proposed between surface properties and sorption performance. An apparent association was observed between pzc and the experimental sorption uptake under near-neutral conditions.

The inclusion of the commercial activated carbon within the correlation analysis further supports this trend, as its intermediate pzc value resulted in sorption and diffusion behavior located between ZnCl_2_- and H_3_PO_4_-activated materials. Conversely, total acidity showed a weaker or even negative correlation with sorption under near-neutral conditions, suggesting that excessive surface acidity promotes an electrostatic repulsion with anionic model molecules. Figure 3 shows a suggested tendency when combining pzc and functional groups distribution, which also indicates the relationship includes stricture, property performance.

From inspection of Figure 4a, it is possible to see a relationship between the intraparticle diffusion rate constant (k_id_) and the surface chemical properties of the studied materials. A moderate dependence of k_id_ on both pzc and total acidity can be observed, although the correlation is not linear, reflecting the complexity of the sorption process. It is also possible to see that materials with near-neutral pzc values, particularly ZnCl_2_-activated carbons, tend to exhibit higher k_id_ values, indicating enhanced mass transfer and more efficient intraparticle diffusion. This behavior is consistent with their more balanced surface charge, which minimizes electrostatic repulsion with ionizable model molecules under near-neutral conditions. Conversely, H_3_PO_4_-activated materials, characterized by low pzc and high surface acidity, display intermediate k_id_ values, suggesting that strong sorbate-surface interactions may partially hinder diffusion processes. This is further supported by Figure 4b, where materials with lower total acidity tend to exhibit slightly improved diffusion behavior.

Biochars showed a more dispersed behavior that suggests their sorption kinetics are mainly influenced by a combination of surface heterogeneity and less developed pore structures; these results suggest that intraparticle diffusion is not governed solely by textural properties but is strongly influenced by surface chemistry, particularly the balanced functional groups.

Additional information is included in Supplementary Material; Figure S1 shows the representative sorption kinetics under different pH regimes for naproxen, ibuprofen, and diclofenac, while Figure S2 shows the correlation between intraparticle diffusion rate constant (k_id_) and initial pH of carbonaceous materials.

Similar approaches have been reported in the literature, where sorption performance is correlated with physical and chemical descriptors such as surface charge and functional group density [25].

Based on the integrated results, a comprehensive sorption process is proposed; since the precursor determined the initial carbon structure while the activating agent controls the development of surface functional groups and porosity, these properties define the pzc, which is an important parameter that controls the pH drift during the sorption process.

The resulting pH determines the speciation of model molecules, which predominantly controls the interaction sorption process given by π–π interactions, hydrogen bonding, and electrostatic forces; these interactions control both sorption capacity and kinetics. In this sense, the sorption is a dynamic process governed by coupled acid–base and interfacial phenomena rather than the equilibrium process.

### 2.5. Implications and Limitations of the Observed Sorption Behavior

The present results show that the relative sorption performance of the carbon materials varied with the investigated pH conditions and their surface acid–base properties. Therefore, the observed performance is discussed here specifically within the experimental conditions evaluated. The experimental results suggest that the sorption system do not operate at constant pH but rather evolve toward a near-neutral condition regardless of the initial pH. Consequently, materials must be evaluated not only based on their maximum sorption capacity but also on their ability to maintain performance across dynamic pH environments.

Some ZnCl_2_-activated carbons maintained comparatively high experimental NSAID uptake across the investigated pH. This behavior is attributed to their near neutral pzc and balanced distribution of surface functional groups, which minimizes electrostatic repulsion with anionic pharmaceuticals under environmentally relevant conditions. The improved sorption is likely the result of the combined contribution of aromatic interactions, pore accessibility, hydrogen bonding, and reduced electrostatic repulsion. In contrast, H_3_PO_4_-activated carbons showed strong pH-dependent performance, with enhanced sorption under acidic conditions but a significant decline at neutral pH due to their highly acidic surface charge.

This observation aligns with previous studies emphasizing that sorbents with pzc close to the operating pH exhibit improved stability and sorption efficiency, particularly for ionizable compounds [37]. Furthermore, the pH-dependent sorption has been widely discussed in the context of pharmaceuticals removal, where electrostatic interactions and speciation strongly influence the sorption performance [38,39,40].

From a design perspective, our findings suggest that sorbents intended for real wastewater treatment applications should be optimized to improve robustness rather than peak performance under controlled conditions. In this regard, materials with moderate surface acidity and pzc values close to neutral are more likely to perform consistently in complex and real environments. Further evaluation in multicomponent and real wastewater matrices, together with regeneration and reuse studies, will be required before their practical treatment performance can be established.

Our result showed that the choice of activating agent plays an important role in tailoring the sorption properties of carbonaceous materials; specifically, ZnCl_2_ and H_3_PO_4_ activation routes generate different surface chemistries, which directly influence sorption process and efficiency.

H_3_PO_4_ activation leads to materials rich in oxygenated acidic groups, which favor hydrogen bonding and polar interactions. These materials are particularly effective under acidic conditions and could be suitable for applications involving acidic effluents or neutral organic contaminants.

In contrast, ZnCl_2_ activation produces materials with mode developed aromatic structures and balanced surface chemistry, promoting π–π interactions, and reducing electrostatic repulsion [9]. Therefore, the corresponding activated materials are more effective for the sorption of aromatic and ionizable compounds under near-neutral conditions, as suggested by our results in this study.

Thus, the selection of activating agent should not be based solely on surface area or yield but on its ability to tailor surface chemistry in concordance with the physicochemical properties of the target pollutant.

It is well known that real effluents are typically unbuffered and subject to continuous fluctuations in pH due to biological activity, chemical reactions, and the presence of multiple contaminants. The present non-buffered experiments provide information on the extent to which carbon surface acid–base properties can be associated with changes between the initially adjusted and final solution pH. Further experiments in multicomponent and real wastewater matrices will be required to establish the relevance of these effects under practical treatment conditions.

Previous studies have highlighted that pH fluctuations can significantly alter sorption capacity and mechanism, particularly for ionizable compounds [18,41]. However, these effects are rarely quantified or incorporated into sorbent design strategies.

The findings of this work suggest that evaluating sorbents under non-buffered conditions provides a more realistic assessment of their performance. Materials that exhibit apparent acid–base buffering effects or stable sorption across varying pH conditions are better suited for practical applications. Moreover, the observed convergence toward neutral pH may have implications for downstream processes, such as biological treatment or advanced oxidation, where pH plays a critical role.

The mechanistic interpretation of the present results should be considered within the limits of the available characterization. Surface acid–base groups and PZC were evaluated directly, while additional morphological, spectroscopic, and textural characteristics of the biomass-derived activated carbons have been reported in previous studies [9,10,19]. However, no post-sorption FTIR/XPS or textural characterization was performed for the NSAID-loaded materials. Therefore, specific molecular interactions such as π–π interactions, hydrogen bonding, and pore filling cannot be experimentally distinguished or quantified from the present dataset. These interactions are considered only as plausible contributions based on NSAID speciation, carbon surface chemistry, and the previous literature.

## 3. Materials and Methods

Coconut shell (C) and orange peel (O) residues were collected from local agroindustrial sources, washed thoroughly with deionized water to remove adhering impurities, and dried at 105 °C for 24 h.

Coconut shell and orange waste were used as lignocellulosic precursors for the preparation of the carbon materials. The biomass-derived adsorbents investigated in this work correspond to materials prepared according to the previously published [9,10,19]. Briefly, the materials comprised non-activated biochars (BC and BO) and chemically activated carbons prepared using ZnCl_2_ (CZ and OZ) or H_3_PO_4_ (CP and OP). The complete preparation procedures, including precursor pretreatment, impregnation conditions, activating-agent concentration or mass ratio, carbonization temperature and time, N_2_ atmosphere and flow, washing, and drying conditions, are reported in the cited methodological studies [10,19].

Biochars derived from coconut shell (BC) and orange peel (BO) were prepared via slow carbonization in a tubular furnace under limited oxygen conditions. The biomass was heated to 300 °C (coconut shell) and 600 °C (orange peel) at a heating rate of 10 °C min^−1^ and maintained for 1 h. After cooling to room temperature under inert conditions, the resulting biochars were collected, washed, and dried.

Phosphoric acid (H_3_PO_4_, 85% wt. from J.T. Baker, Phillipsburg, NJ, USA) and zinc chloride (ZnCl_2_, analytical grade from Karal, León, Mexico) were used as activating agents without further purification. A commercial vegetable-based activated carbon (CAC) was used as a reference material.

The biomass-derived activated carbons investigated in the present work have been previously characterized in terms of their physicochemical properties. The H_3_PO_4_-activated orange peel and coconut shell carbons were previously analyzed by FTIR, SEM-EDS, Boehm titration, potentiometric titration, and PZC measurements [9,10], while the corresponding ZnCl_2_-activated materials were subsequently characterized using comparable methods [9]. N_2_ adsorption–desorption/BET characterization of the activated carbons derived from both precursors and prepared using the two activating agents has also been reported previously [9,10]. These previous characterizations are used herein only as physicochemical background of the adsorbents and not as direct evidence of molecular interactions occurring during NSAID sorption.

**Figure 5:**
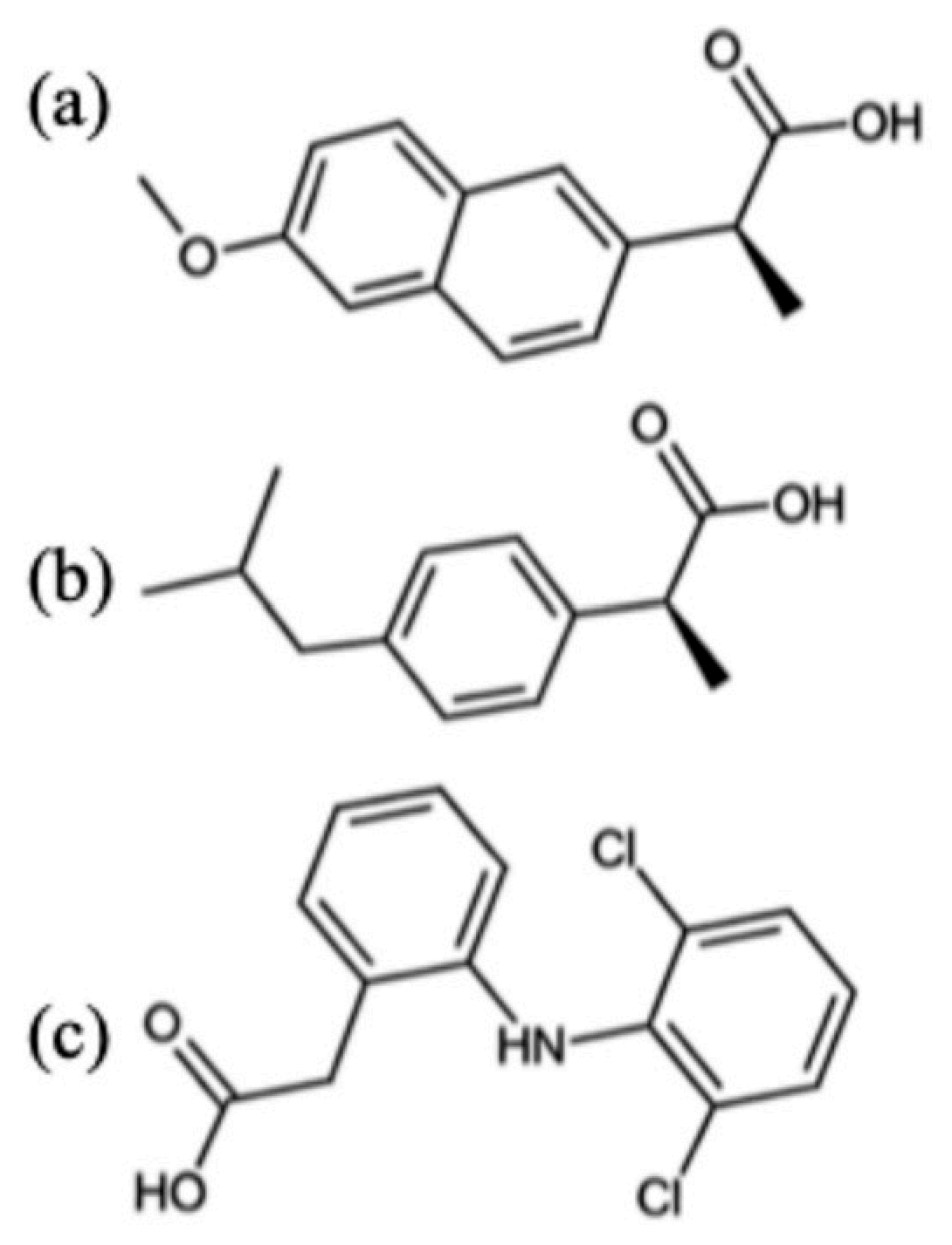
Comparison of molecular structures of (**a**) naproxen, (**b**) ibuprofen, and (**c**) diclofenac.

Naproxen, ibuprofen, and diclofenac sodium (analytical grade, from Sigma-Aldrich, St. Louis, MO, USA) were used as model pharmaceutical compounds. All solutions were prepared using deionized water. The representation of the molecular structures are presented in Figure 5.

The surface functional groups were quantified using Boehm titration, which allows for the determination of acidic groups (carboxylic, lactone, and phenolic) and basic sites. A sample of carbon material was equilibrated with NaOH (from J.T. Baker, Phillipsburg, NJ, USA), Na_2_CO_3_ (from J.T. Baker, Phillipsburg, NJ, USA), NaHCO_3_ (from J.T. Baker, Phillipsburg, NJ, USA), or HCl (from J.T. Baker, Phillipsburg, NJ, USA) solutions of known concentration. After equilibration, aliquots were titrated with standardized solutions to determine the consumption of each reagent. The concentrations of functional groups were calculated according to established methodologies [9,10].

The point of zero charge (pzc) was determined using the pH drift method. A series of NaCl solutions (0.01 M) were adjusted to initial pH values between 2 and 12. Carbon samples (0.1 g) were added to 50 mL of each solution and equilibrated for 24 h. Additionally, a potentiometric titration was carried out to assess the surface’s basicity or acidity of the samples [9,10].

Sorption experiments were conducted in batch mode using 100 mL of pharmaceutical solutions at a fixed initial concentration of 0.3 mM for naproxen and ibuprofen and 0.07 mM for diclofenac, considering the dilution in water of the molecule. A mass of 20 mg of sorbent material was added to 40 mL solution, and the suspensions were agitated at constant temperature. The initial pH values were adjusted to 3, 5, 7, and 9 using dilute HCl or NaOH solutions. The pH was measured at the beginning and at the final sampling time to quantify the initial-to-final pH shift. The solution pH was measured at the beginning of each sorption experiment pH_i_ and at the final sampling time pH_f_. The net pH shift was expressed as indicated in Equation (1):

ΔpH = pH_f_ − pH_i_ (1)

where positive and negative ΔpH values indicate an increase and decrease in solution pH during the experimental contact period, respectively.

Samples were taken at predetermined time intervals, filtered, and analyzed using UV-Vis spectroscopy (Thermo Scientific, Genesis G10S, Waltham, MA, USA) at 223 nm for ibuprofen, 275 nm for diclofenac, and 230 nm for naproxen, individually. Sorbent blanks containing the corresponding carbon material under otherwise identical experimental conditions but without pharmaceutical were used to account for background absorbance associated with the sorbent.

The sorption capacity (*q_t_*, mg/g) was calculated using Equation (2), where *C*_0_ and *C_t_* are the initial and time-dependent concentrations (mg/L), *V* is the solution volume (L), and *m* is the sorbent mass (g).

(2) $$q_{t}=\frac{\left(C_{0}-C_{t}\right)V}{m}$$

The adsorption capacity calculated from Equation (1) represents the amount of pharmaceutical adsorbed per unit mass of sorbent under the specific initial concentration, pH, and experimental conditions investigated. Because adsorption experiments were conducted at a single initial pharmaceutical concentration, these values should not be interpreted as maximum adsorption capacities *q_max_*. Determination of *q_max_* would require equilibrium adsorption experiments over a range of initial concentrations. All sorption experiments were performed in triplicate using independent containers. Each model molecule was investigated independently.

For each sorbent–pharmaceutical system, operational equilibrium was considered to have been reached when the measured pharmaceutical concentration no longer changed with increasing contact time under the experimental conditions. The corresponding equilibrium uptake *q_e_* therefore represents the amount adsorbed at the concentration plateau observed for the fixed initial pharmaceutical concentration investigated.

Kinetic data were analyzed using time-dependent sorption profiles (*q_t_* vs. *t*). Due to the observed non-stationary conditions associated with pH drift, classical pseudo-first-order and pseudo-second-order models were complemented with diffusion-based analysis. Kinetic data were evaluated using the experimental time-dependent sorption profiles *q_t_* vs. *t*. The Elovich equation and the Weber-Morris (see Equation (3)) intraparticle diffusion model were used as descriptive tools to compare the apparent kinetic behavior among the investigated sorbent–pharmaceutical systems. Because the first experimental sampling point was collected at 0.5 h, these analyses do not resolve the initial uptake period and were not used to assign a unique rate-controlling process.

(3) $$q_{t}=k_{id}t^{1/2}+C$$

where *k_id_* refers to intraparticle diffusion rate constant and C commonly associated with boundary layer effects. Multilinearity in the Weber–Morris representation was used only to identify changes in the apparent uptake regime over the experimental time range. Such behavior does not, by itself, establish the rate-controlling mechanism [42].

Accordingly, the Weber–Morris analysis was used here as a descriptive rather than definitive mechanistic tool. Complementary to Weber–Morris, the Elovich equation was used as an empirical model to describe the time-dependent sorption profiles, where α represents the initial apparent sorption-rate parameter and β is an empirical parameter related to the variation of the sorption rate with surface coverage. In the present study, Elovich fitting is not interpreted as direct evidence of chemisorption, surface activation-energy distributions, or a specific molecular mechanism.

## 4. Conclusions

This study evaluated the sorption of naproxen, ibuprofen, and diclofenac onto agroindustrial-derived carbons obtained from coconut shell and orange peel, focusing on the influence of activation method, surface acid–base properties, and the comparison between initial and final pH values during the sorption process. The results provide an integrated interpretation of the associations among surface chemistry, solution pH, and sorption behavior.

The activating agent was identified as the primary determinant of surface chemistry and acid–base properties of the prepared materials. H_3_PO_4_ activation produced highly acidic materials with lower pzc values, whereas ZnCl_2_ activation yielded materials with pzc values closes and lower net acidity. These differences were associated with variations in sorption performance and pH evolution.

Although the experiments were initiated at controlled pH values, the solution pH changed during the sorption process. Those systems that initially were adjusted to pH 5 to 9 generally shifted toward near-neutral values, whereas strong acidic systems remain within an acidic range. This behavior was influenced by surface acid–base reactions, proton exchange, and possible residual activating species.

The sorption response varied with the activation route, precursor, and pH conditions. H_3_PO_4_- and ZnCl_2_-activated materials exhibited distinct surface acid–base characteristics and PZC values, which were associated with differences in the observed NSAID uptake. Although electrostatic and non-electrostatic interactions may jointly contribute to sorption, the present dataset does not permit definitive assignment of individual molecular mechanisms.

Previous physicochemical characterization of these biomass-derived carbons provides complementary information on their morphology, chemical functionalities, and textural properties; however, post-sorption spectroscopic and textural characterization would be required to directly establish specific NSAID–surface interactions and adsorption-induced changes.

Under the conditions evaluated, some ZnCl_2_-activated carbons exhibited comparatively high NSAID uptake. This behavior was associated with differences in their measured surface acid–base properties and PZC, although the individual contributions of electrostatic, textural, and other molecular interactions cannot be resolved from the present dataset.

Classical pseudo-order models provided limited descriptions of the experimental profiles. This limitation may be related to rapid initial uptake, evolving pH surface heterogeneity, and the simultaneous contribution of several mass-transport processes. The experimental profiles showed rapid uptake before or by the first measured interval, followed by smaller concentration changes at longer contact times. Because the initial uptake period was not experimentally resolved, the kinetic models were interpreted only as empirical descriptors. Multilinear Weber–Morris behavior and non-zero intercepts indicate that intraparticle diffusion cannot be considered the sole rate-limiting contribution, while Elovich fitting does not establish a specific molecular mechanism.

Sorption performance showed an apparent association with pzc and surface functional groups distribution, supporting a preliminary performance relationship. Among the material evaluated the ZnCl_2_-activated coconut shell carbon showed the highest overall sorption response under the experimental conditions investigated. Because the experiments were conducted at a fixed initial pharmaceutical concentration, the observed sorption values do not represent maximum adsorption capacities. Accordingly, determination of equilibrium isotherms and *q_max_* values will be necessary in future studies to quantitatively compare the saturation capacities of these materials.

Finally, the sorption framework provides a preliminary basis for designing carbonaceous sorbents, emphasizing the importance of tailoring surface chemistry in relation to the pKa of target contaminants and the expected operating pH. Future design strategies should consider additional chemical properties of interferents in wastewater.

## Figures and Tables

**Table 1:** Surface chemical properties, acid–base behavior, and interpretation of potentiometric equivalence point of the carbon materials.

Precursor	COOH (mmol/g)	Lactones (mmol/g)	Phenolics (mmol/g)	Acid Groups (mmol/g)	Basic Groups (mmol/g)	Net Acidity Index (mmol/g)	PZC	Equivalence Point
Commercial activated carbon as reference material								
CAC	1.6	0.4	0.103	2.103	2.07	0.033	5.7 ± 0.1	1.1 ± 0.1
Biochar (carbonized materials without chemical activation)								
BO	2.68	0.22	0.754	3.654	3.6	0.054	7.2 ± 0.09	1.08 ± 0.03
BC	2.84	0.144	0.694	3.678	3.64	0.038	8.0 ± 0.1	1.26 ± 0.05
ZnCl_2_ activated carbons								
OZ	2.2	0.26	1.189	3.649	2.48	1.169	6.8 ± 0.07	1.26 ± 0.1
CZ	1.92	0.54	1.192	3.652	2.68	0.972	7.5 ± 0.04	2.79 ± 0.09
H_3_PO_4_ activated carbons								
OP	0.237	5.073	0.003	5.313	4.89	0.423	2.0 ± 0.07	1.66 ± 0.08
CP	0.116	5.254	0	5.367	4.905	0.381	1.4 ± 0.05	1.48 ± 0.07

Where CAC—commercial vegetal activated carbon, BO—biocarbon from orange peel, BC—biocarbon from coconut shell, OZ—ZnCl_2_-activated carbon from orange peel, CZ—ZnCl_2_-activated carbon from coconut shell, OP—H_3_PO_4_-activated carbon from orange peel, and CP—H_3_PO_4_-activated carbon from coconut shell. Acid groups refers to the sum of carboxylic, lactone, and phenolic groups. The et acidity index refers to the ratio given by acid and basic groups. The equivalence point was calculated from a potentiometric titration. Acid and basic groups showed a standard deviation lower than 5%.

**Table 2:** Comparative kinetic modeling of NSAIDs sorption on carbon materials under different pH conditions.

Material	pH	Best-Fit Model	R^2^ (Range)	Key Parameters	Suggested Descriptive Interpretation Based on Model Fitting
Naproxen					
CAC	7	Weber–Morris	0.92–0.96	k_id_ ≈ 5–8; C ≠ 0	Fast surface sorption followed by diffusion control
CAC	3	Weber–Morris	0.93–0.97	k_id_ ≈ 6–10; C ≠ 0	Enhanced diffusion under acidic conditions
CAC	5	Diffusion-dominated	0.88–0.94	—	Rapid equilibrium limits model fitting
CAC	9	Weber–Morris	0.90–0.95	k_id_ ≈ 4–7	Slight electrostatic limitation
BO	all	Elovich, W-M	0.9–0.96	α high; β moderate	Heterogeneous surface with multi-energy sites
OZ	all	W-M	0.93–0.98	k_id_ ≈ 8–15	Strong intraparticle diffusion contribution
BC	all	Elovich	0.89–0.95	α moderate	Surface heterogeneity dominates
CZ	all	Weber–Morris	0.92–0.97	k_id_ ≈ 7–12	Combined surface + diffusion mechanism
OP	all	Elovich	0.91–0.96	α variable; β high	Chemically controlled sorption on acidic surface
CP	all	Elovich	0.90–0.95	α low; β high	Strong influence of acidic functional groups
Ibuprofen					
CAC	all	Weber–Morris	0.90–0.96	k_id_ ≈ 6–10	Diffusion-controlled sorption
BO	all	Elovich	0.88–0.94	α moderate	Surface heterogeneity dominant
OZ	all	Weber–Morris	0.92–0.97	k_id_ ≈ 10–18	Fast diffusion and favorable pore accessibility
BC	all	Poor fit	<0.90	—	Irregular sorption behavior
CZ	all	Weber–Morris	0.91–0.96	k_id_ ≈ 8–14	Diffusion + surface interaction
OP	all	Elovich	0.89–0.95	α variable	Acidic surface-controlled sorption
CP	all	Elovich	0.89–0.95	β high	Slow sorption due to electrostatic effects
Diclofenac					
CAC	all	Weber–Morris	0.93–0.98	k_id_ ≈ 10–20	Strong diffusion and π–π interactions
BO	all	Elovich	0.91–0.96	α high	Highly heterogeneous sorption sites
OZ	all	Weber–Morris	0.94–0.99	k_id_ ≈ 15–25	Highly efficient diffusion-driven sorption
BC	all	Elovich	0.90–0.95	α moderate	Surface heterogeneity dominates
CZ	all	Weber–Morris	0.93–0.98	k_id_ ≈ 12–22	Combined pore diffusion and surface interaction
OP	all	Elovich	0.91–0.96	β high	Strong acidic interaction control
CP	all	Elovich	0.91–0.96	β high	Slower sorption with electrostatic limitation

## Data Availability

Data is contained within the article or Supplementary Material.

## Supplementary Materials

The following supporting information can be downloaded at https://www.mdpi.com/article/10.3390/molecules31183209/s1, Figure S1: Representative adsorption kinetics under different pH regimes (a) (naproxen), (b) (ibuprofen), (c) (diclofenac). Time-dependent adsorption profiles (qₜ vs t) for representative materials, illustrating differences in adsorption rates and equilibrium behavior. Intraparticle diffusion analysis, (d) (naproxen), (e) (ibuprofen), (f) (diclofenac), of Weber–Morris plots (qₜ vs t^1/2^) for representative materials, showing multi-stage adsorption behavior and identifying the contribution of intraparticle diffusion and boundary layer effects; Figure S2: Correlation between intraparticle diffusion rate constant (k_id_) and initial pH of the carbon materials, for (a) naproxen, (b) ibuprofen), and (c) diclofenac.
