# Supplementary material for: The Role of Surface Chemistry and pH Shifts in the Sorption of Common NSAIDs on Coconut and Orange Waste-Derived Carbon Materials
Source: Molecules. 2026 Sep 11;31(18):3209. doi: 10.3390/molecules31183209 (PMC13609005; doi:10.3390/molecules31183209)
Supplement: Supplementary file 1 [file molecules-31-03209-s001.zip › molecules-4545736-supplementary.pdf]

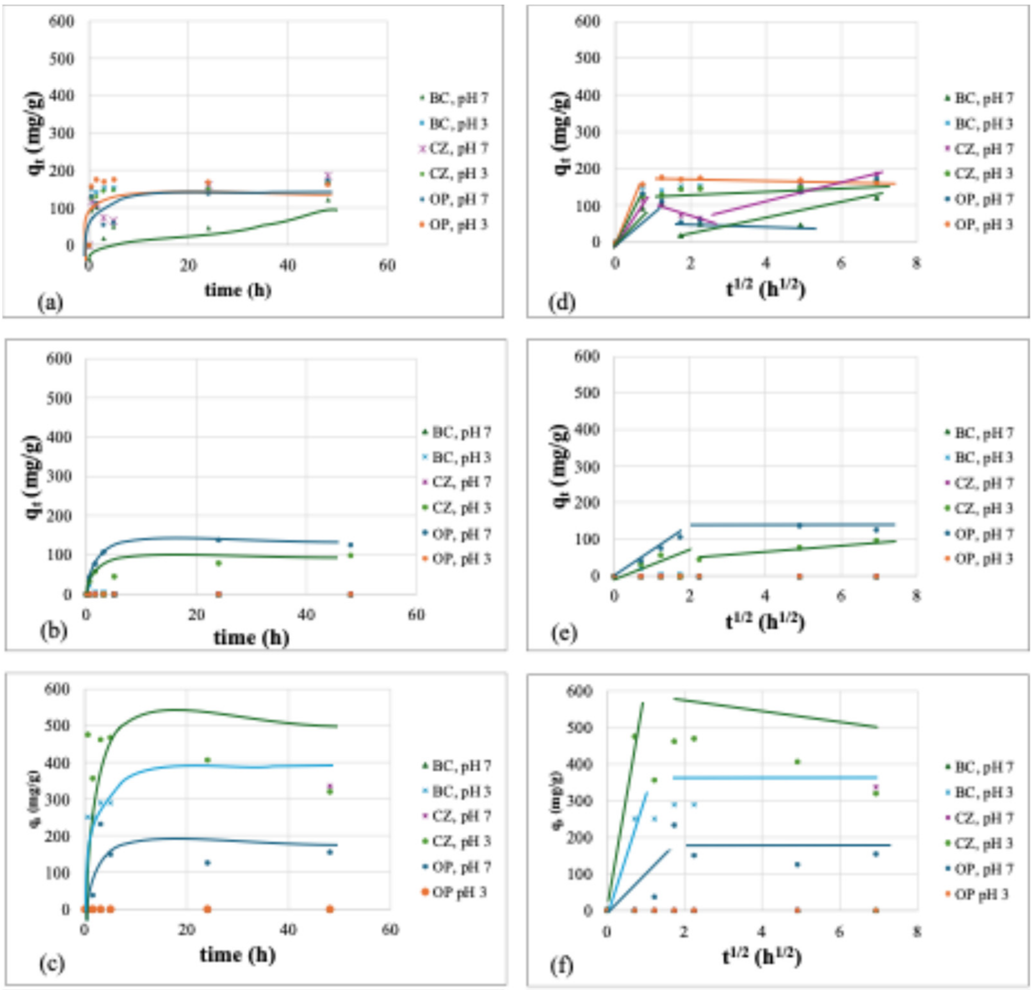

Figure S1. Representative adsorption kinetics under different pH regimes (a) (naproxen), (b) (ibuprofen), (c) (diclofenac). Time-dependent adsorption profiles (q_t vs t) for representative materials, illustrating differences in adsorption rates and equilibrium behavior. Intraparticle diffusion analysis: (d) (naproxen), (e) (ibuprofen), (f) (diclofenac) of Weber–Morris plots (q_t vs $t^{1/2}$) for representative materials, showing multi-stage adsorption behavior and identifying the contribution of intraparticle diffusion and boundary layer effects.

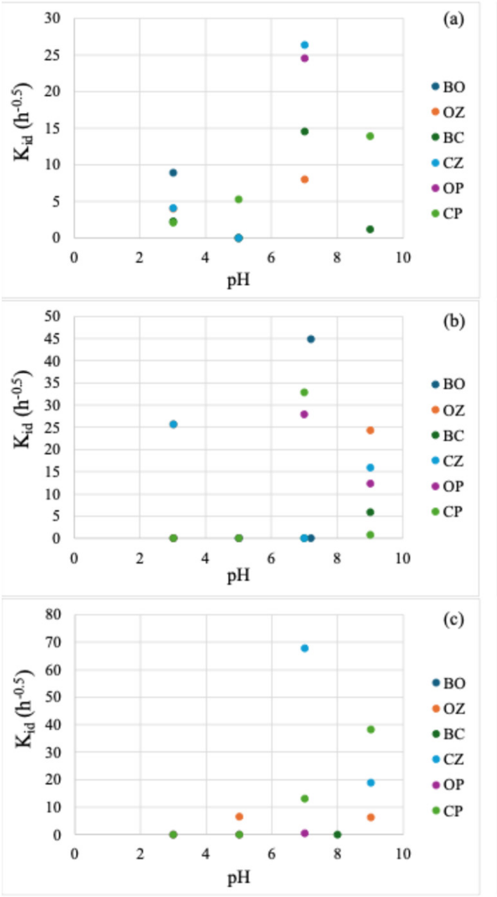

Figure S2. Correlation between intraparticle diffusion rate constant (k_{id}) and initial pH of the carbon materials, for (a) naproxen, (b) ibuprofen), and (c) diclofenac.
